# International Charitable Connections: the Growth in Number, and the Countries of Operation, of English and Welsh Charities Working Overseas

**DOI:** 10.1017/S0047279416000076

**Published:** 2016-07

**Authors:** DAVID CLIFFORD

**Affiliations:** Faculty of Social and Human Sciences, University of Southampton, Southampton, SO17 1BJ, UK email: david.clifford@soton.ac.uk

## Abstract

This paper provides new empirical evidence about English and Welsh charities operating internationally. It answers basic questions unaddressed in existing work: how many charities work overseas, and how has this number changed over time? In which countries do they operate, and what underlies these geographical patterns? It makes use of a unique administrative dataset which records every country in which each charity operates. The results show a sizeable increase in the number of charities working overseas since the mid-1990s. They show that charities are much more likely to work in countries with colonial and linguistic ties to the UK, and less likely to work in countries with high levels of instability or corruption. This considerable geographical unevenness, even after controlling for countries’ population size and poverty, illustrates the importance of supply-side theories and of institutional factors to an understanding of international voluntary activity. The paper also serves to provide a new perspective on international charitable operation: while it is the large development charities that are household names, the results reveal the extent of small-scale ‘grassroots’ registered charitable activity that links people and places internationally, and the extent of activity in ‘developed’ as well as ‘developing’ country contexts.

## Introduction: a shortage of research within social policy

International voluntary organisations have been relatively neglected within the study of social policy. This reflects a wider historical ‘double knowledge gap’ within social policy research (Lewis, 2013). First, reflecting its intellectual heritage, research has tended to focus on national social policy in industrialised country contexts. Second, research has tended to focus on the welfare state, with less emphasis on the role of non-state actors. However, the importance of research on international voluntary organisations has recently been highlighted by an emerging move – as part of a conversation between social policy and development studies – towards considering social policy within ‘developing’ country contexts, where the role of domestic and international ‘non-governmental organisations’ in the provision of welfare cannot be ignored (Gough and Wood, 2004; Surender and Walker, 2013). The importance of research into international voluntary organisations has been further underlined by the rise of the field of global social policy, which has highlighted the role of actors whose activities transcend the nation state. This includes not only the activities of elite international financial institutions but also those of a wide range of actors, including large and small voluntary organisations, that link people and places internationally (Yeates, 2008; Yeates and Holden, 2009).

These developments provide further strength to calls to end the ‘parallel worlds’ of separate research which sees ‘domestic’ ‘voluntary organisations’ (or ‘nonprofit organisations’) increasingly studied within social policy but the study of international ‘NGOs’ confined largely to development studies (Lewis, 2014). This de facto separation occurs despite common ‘structural/operational’ approaches to definition: voluntary organisations are formal organisations (with internal structure and meaningful boundaries) which are self-governing, independent of government, not profit-distributing, and voluntary, while NGOs are often understood as the subset of voluntary organisations engaged in humanitarian or development work (Salamon and Anheier, 1992; Kendall and Knapp, 1993; Vakil, 1997; Lewis, 2013). This separation has persisted, as Lewis (2014) points out, despite common themes in the respective literatures surrounding, for example, accountability, effectiveness, and the nature of the relationship with the state; despite common interests in concepts like ‘social capital’ and ‘civil society’; and despite patterns of globalisation which problematise binary distinctions between ‘developed’ and ‘developing’ countries. The separation has served to hinder the potential for learning across the two literatures. Importantly, it may also have served to divert scholarly attention from the many international voluntary organisations that are not ‘NGOs’ in the sense that they do not work in development or in ‘developing’ country contexts.

There has been a particular shortage of research which has been able to provide basic empirical evidence about the trends in the number of voluntary organisations operating internationally, and about the geographical patterns in their operation. First, in terms of trends, there are accounts that the extensity and intensity of contemporary global networks (Held *et al.*, 1999; Bebbington and Kothari, 2006), reflected for example in advances in communication and increased international travel, have facilitated an increase in the number of international ‘citizen initiatives’ – small-scale ‘grassroots’ voluntary organisations which directly involve non-development professionals in the provision of goods and services overseas (Develtere and De Bruyn, 2009; Schnable, 2014; Kinsbergen *et al.*, 2013). However, there is little empirical evidence with which to examine this apparent trend: since income across the sector is dominated by large development organisations, trends in aggregate voluntary income for international causes – documented, for example, by Atkinson *et al.* (2012) – provide no insight into the number of smaller international organisations; the Yearbook for International Organisations has historical data on numbers of organisations, but excludes many smaller organisations since it is intended to include only those oriented to three or more countries.

Second, in terms of geographical patterns in the operation of international voluntary organisations, there is growing recognition of the importance of improving the scant evidence base (Watkins *et al.*, 2012). This is particularly the case for organisations working in development, given a prominent debate about priorities for aid. Recently the bilateral aid review, in which country priorities were reassessed, heralded significant changes in the allocation of UK official aid and the end of financial assistance to a number of countries (DFID, 2011). Many voluntary organisations, as well as official agencies, have been reviewing their priorities: Dame Anne Owers, the Chair of Christian Aid, acknowledged that “Christian Aid and the wider development community have big decisions to make about where we work, and how” (Christian Aid, 2011). However, while geographical patterns in official (government) development assistance (ODA) are well monitored, much less is known about the geography of operation of international voluntary organisations (Agg, 2006; Hénon, 2014; International Development Committee, 2012). An emerging literature has started to examine the aid allocation of international NGOs (Nunnenkamp *et al.*, 2009; Dreher *et al.*, 2010; Dreher *et al.*, 2012; Büthe *et al.*, 2012), but none of these studies focus on UK-based international voluntary organisations.

Therefore, given the lack of existing research, this paper examines the international activity of voluntary organisations registered as charities in England and Wales. Basic questions remain unanswered in existing empirical work: for example, how many charities operate internationally, and how has this number changed over time? Has there been an increase in the number of small-scale ‘grassroots’ international charities? What is the geographical pattern in the country of operation of international charities? Are certain countries distinctive in terms of the high, or low, number of charities working there? Does charitable operation tend to focus on countries with poor governance, or are charities less likely to work in countries with high levels of instability and/or corruption? Are charities more likely to work in countries with historical colonial links to the UK? What is the relationship with the pattern of UK ODA: does charitable operation tend to coincide with, or differ from, government priorities? This paper answers these questions for the first time.

We adopt a distinctive empirical approach. We examine charitable operation across all countries, and therefore are able to move away from a restrictive focus on either ‘developing’ or ‘developed’ country contexts, often characteristic of the ‘parallel worlds’ of research into voluntary organisations and NGOs respectively (Lewis, 2014). We include not only the large development organisations, which are typically the focus of the few existing studies in the emerging literature on the aid allocation of NGOs (Koch, 2009; Büthe *et al.*, 2012; Dreher *et al.*, 2012), but also we are able to provide evidence about smaller ‘grassroots’ international voluntary organisations. This is important because it is in keeping with global social policy's interest in the dense network of international connections of ‘non-elite’, as well as elite, actors (Yeates and Holden, 2009).

## Data and method

The paper makes use of a unique administrative dataset from the Charity Commission (CC). The CC registers and regulates charities – voluntary organisations that benefit the public in a way that the law says is charitable – in England and Wales. Through the returns that each of these charities are required to complete annually, the CC collects information on every country in which each charity operates. This is a mandatory field, so is completed by all charities. The information has not yet been used in academic research.

The main analysis of this paper is based on CC data from 2014. The total number of registered charities in England and Wales in 2014 is c.163,000. The analysis in this paper shows that, of this total, c.16,500 indicate that they operate in at least one country or territory outside the UK. Since we have information not only on the population of currently registered organisations, but also on all of those that have registered and dissolved in our analysis period, this paper is also able to present trends in the number of registered charities operating internationally between 1995 and 2014^1^. Further details about the CC data, including a discussion of data quality and the steps involved in the data preparation, are provided in section 1 of the online supplementary material.

We link the data on the international operation of charities to relevant country-level covariate data. Table 1 presents descriptive statistics on the distribution of countries according to these covariates. The World Bank's (2012) country classification identifies high income countries and those in different regions. The Worldwide Governance Indicators (WGI) (Kaufman *et al.*, 2011) provide measures of political stability and absence of violence/terrorism, and of control of corruption. We include measures of the links between the UK and overseas: whether the country is a former British territory (Mayer and Zignago, 2011); whether the country has English as an official and/or common^2^ language (Mayer and Zignago, 2011); and 2011 census information on the number of people by their country of birth for residents of England and Wales (ONS, 2013). To compare current patterns of official UK aid with patterns of overseas charitable operation, we link in information on bilateral priorities for official aid from the Department for International Development (DFID, 2011). We also link in data on total population size (World Bank, 2013). We use the recently developed Multidimensional Poverty Index (MPI) (Alkire *et al.*, 2014) as a source of data on the number of people living in poverty in a country, reflecting an understanding of poverty as a multi-dimensional concept not well described by income alone.

**TABLE 1.** Number of countries by country-level covariates

	N	%
*Region*		
High income	57	*28*
East Asia & Pacific	23	*11*
Europe and Central Asia	23	*11*
Latin America & Caribbean	30	*15*
Middle East & North Africa	13	*6*
South Asia	8	*4*
Sub-Saharan Africa	47	*23*
*Governance: instability*		
Decile 2–9 of WGI distribution	180	*90*
Top decile	21	*10*
*Governance: corruption*		
Decile 2–9 of WGI distribution	180	*90*
Top decile	21	*10*
*History: British Empire*		
Not former British territory	133	*66*
Former British territory	68	*34*
*History: No. of E and W residents born in country*		
Less than 50k	168	*84*
50k-100k	14	*7*
More than 100k	19	*9*
*History: official / common spoken languages*		
Doesn't include English	134	*67*
Includes English	67	*33*
*Government: DFID priority for official aid*		
Not focus country	174	*87*
Focus country	27	*13*
Total	201	*100*

*Notes:* The regional covariate treats high-income countries within these regions as a separate category.

When examining geographical patterns in the overseas operation of charities we restrict analysis to charities that operate outside the UK, considering the operation of 16,274 charities across 201 countries^3^. We organise our data into 3,271,074 rows defined by unique combinations of charity and country. We generate a 0/1 indicator variable which, for each of these row combinations, indicates whether or not that charity operates in that country: for each charity, countries are coded 1 where a charity reports operation and 0 where no operation is reported. The mean number of countries in which a charity operates is 4.7. Therefore, across the population of charities that operate overseas, the average probability π of a charity operating in any given overseas country is 4.7/201=0.023^4^. Using the 0/1 indicator variable as our outcome, we use logistic regression to examine how this probability π_*i*_ varies according to our covariates: 
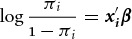
 where the *i* observations are defined by the 3,271,074 unique combinations of charity and country, 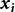 is a vector of covariates and 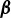 is a vector of coefficients. Our main interest is in assessing how overseas charitable operation varies according to seven country-level covariates of interest (listed in Table 1). However we also include in our regression a charity-level covariate describing the geographical scope of the charity (whether it operates in one country/2–9 countries/more than 10 countries). We also control for country population size given that, on average, more charities work in countries with bigger populations^5^.

The analysis in this paper has its limitations. It focuses on charities registered in England and Wales that work internationally. It does not consider charities ‘excepted’ or ‘exempted’ from registration with the Charity Commission; those diasporic organisations, including informal groups and mutual funds, with links to their countries of origin but which are not registered charities (see, for example, Van Hear *et al.*, 2004); and noncharitable civil society organisations including mutuals and social enterprises. It does not consider individuals’ remittances which, after foreign direct investment, represent the largest source of external finance for developing countries (see Solimano, 2005), or the work of ‘free-floating altruists’ not connected with an organisation. Therefore, while the paper illustrates the activity of registered charitable organisations that operate overseas – including that of many ‘grassroots’ organisations – this represents only a partial perspective on the nature of philanthropic activity that links people and places internationally.

The paper does not seek to provide insight into the sum of charitable activity in a particular overseas country, since it does not include the activity of ‘domestic’ organisations or of international organisations registered in other countries. It focuses on the ‘selection’ rather than ‘allocation’ stage – whether or not a charity works in a particular country, rather than the share of resources allocated to operation in that country^6^. It focuses on the country level, not on patterns in charitable activity at the sub-national level.

## Results

In total 16,502 charities^7^ indicate that they operate in at least one country or territory outside of the UK^8^. This represents a significant proportion – 10 per cent – of the population of c.163,000 registered charities in England and Wales in March 2014. The total of c.16,500 includes a small number of large organisations, including c.200 with an income of more than £10m, and c.1,000 with an income of more than £1m. However, and notably, the majority of charities operating overseas are small in size. Around a third (34 per cent) have an annual income under £10,000; nearly three quarters (73 per cent) have an income under £100,000^9^. These small organisations draw on significant voluntary resources: there are 54,000 trustees involved in running those overseas charities with an income under £100,000. Most charities operating overseas have a limited geographical scope. Indeed, more than half of charities working overseas (9,050; 55 per cent) operate in just one country; around a third (5,748; 35 per cent) operate in between 2 and 9 countries, while 10 per cent (1,704) operate in 10 countries or more. As expected, charities with a smaller income tend to have a more restricted geographical scope (Table 2). While most overseas charities (9,792, or 59 per cent) operate exclusively within countries that are classed as eligible for ODA by the OECD, a significant fraction (41 per cent) operate in ‘developed’ country contexts – either in addition to operation within ODA-eligible countries (3,348 charities; 20 per cent of the total) or exclusively in non-ODA eligible countries (3,362; 20 per cent).

**TABLE 2.** Number of charities working internationally, by annual income (£) and geographical scope

	Geographical scope (number of countries)			
	One	2–9	10+	Total
Under £10k	2,926	1,430	270	4,626
	*(63)*	*(31)*	*(6)*	
£10k–£100k	3,137	1,889	353	5,379
	*(58)*	*(35)*	*(7)*	
£100k–£1m	1,190	1,063	380	2,633
	*(45)*	*(40)*	*(14)*	
£1m –£10m	245	340	263	848
	*(29)*	*(40)*	*(31)*	
£10m+	33	63	101	197
	*(17)*	*(32)*	*(51)*	
Missing income	1,519	963	337	2,819
	*(54)*	*(34)*	*(12)*	
Total	9,050	5,748	1,704	16,502
	*(55)*	*(35)*	*(10)*	

*Notes:* Row percentages in brackets. Annual income in 2012.

*Source*: author's analysis

### Trends over time

**Figure 1.**
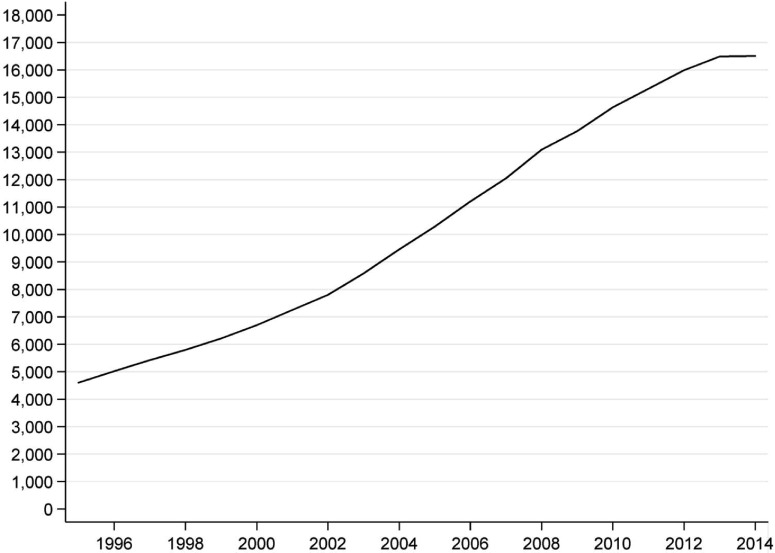
Trend in the number of English and Welsh charities operating internationally

*Source*: author's analysis

**Figure 2.**
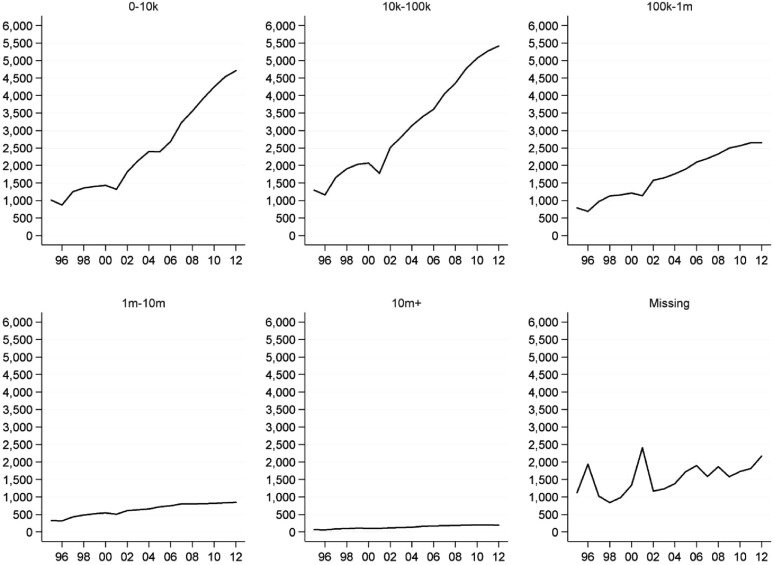
Trend in the number of English and Welsh charities operating internationally, by size (income, £)

*Notes:* Income adjusted for inflation using the Retail Price Index (RPIX). ‘Missing’: missing income data *Source*: author's analysis

The results indicate a sizeable increase in the number of registered charities operating overseas: there are 3.6 times more charities working internationally in 2014 (16,502) than in 1995 (4,599) (Figure 1). Importantly, this is during a period in which the total number of registered charities has remained at around 160,000 throughout, so the share of charities that operate internationally has also increased sizeably. Figure 2 shows the trend disaggregated according to the size of charity, in terms of headline income^10^. There has been an increase in the number of charities working internationally across the whole size distribution. However, in absolute and in relative terms, there has been a more significant increase in the number of small charities than in the number of large charities: there are 4.7 times more charities sized £0–10k in 2012 than in 1995 (4,709/1,007); 4.2 times more charities sized £10k–100k (5,412/1,297); and 3.1 times the number of very large charities (£10m+) (198/63).

### Patterns in country of operation: relationship with covariates

Across the population of 16,274 charities and 201 countries as a whole, the average predicted probability of a charity working in any given overseas country is 0.023 (2.3 per cent). However the results from the logistic regression models show considerable variation across countries in the likelihood of charitable operation. In models A1–A7 we consider patterns according to each of our country-level covariates in turn. While we use the logit link, which models the log-odds, we present the results in terms of predicted probabilities. In each case, the predicted probabilities are calculated for different levels of the covariate of interest while holding other variables in the model, country population size and the geographical scope of the charity, constant at the observed sample values. Then the average of these predicted probabilities is taken across the sample observations. Table 3 collates the results, presenting the average predicted probability of a charity working in any given overseas country by covariate characteristics. The probability of any given charity operating in any given country remains low, conditional on covariates. For example the average predicted probability of a charity working in a given country is 3.7 per cent if the country is a former British territory and 1.7 per cent if the country is not a former British territory. However it is the relative risks (RR: ratios of the probabilities across the different levels of a covariate), rather than the probabilities themselves, that are of particular substantive interest. **TABLE 3.** Logistic regression results (Models A1-A2; considering all charities that operate outside the UK): average predicted probability of a charity working in any given overseas country, by covariate characteristics

	A1		A2	

*Region*				
High income	0.028	*(0.028 - 0.028)*		
East Asia & Pacific	0.012	*(0.012 - 0.013)*		
Europe and Central Asia	0.017	*(0.017 - 0.018)*		
Latin America & Caribbean	0.014	*(0.014 - 0.014)*		
Middle East & North Africa	0.013	*(0.013 - 0.014)*		
South Asia	0.032	*(0.031 - 0.032)*		
Sub-Saharan Africa	0.033	*(0.032 - 0.033)*		
				
*Governance: instability*				
Decile 2–9 of WGI distribution			0.024	*(0.024 - 0.024)*
Top decile			0.021	*(0.020 - 0.021)*
				
*Governance: corruption*				
Decile 2–9 of WGI distribution				
Top decile				
				
*History: British Empire*				
Not former British territory				
Former British territory				
				
*History: No. of E and W residents born in country*				
Less than 50k				
50k-100k				
More than 100k				
				
*History: official / common spoken languages*				
Doesn't include English				
Includes English				
				
*Government: DFID priority for official aid*				
Not focus country				
Focus country				
				
*Geographical scope of charity* (charity-level)				
One country	0.005	*(0.005-0.005)*	0.005	*(0.005-0.005)*
2-9 countries	0.019	*(0.019-0.019)*	0.019	*(0.019-0.019)*
10 countries +	0.146	*(0.144-0.147)*	0.146	*(0.144-0.147)*
				
*N*	*3,271,074*		*3,271,074*	

*Notes*: All models also include controls for the logarithm of the country population size (main effect and squared terms). Number of observations=201 countries × 16,274 charities= 3,271,074. 95% CI in brackets.

*Source*: author's analysis

**TABLE 3 (cont.):** Logistic regression results (Models A3-A4; considering all charities that operate outside the UK): average predicted probability of a charity working in any given overseas country, by covariate characteristics

	A3		A4	

*Region*				
High income				
East Asia & Pacific				
Europe and Central Asia				
Latin America & Caribbean				
Middle East & North Africa				
South Asia				
Sub-Saharan Africa				
				
*Governance: instability*				
Decile 2–9 of WGI distribution				
Top decile				
				
*Governance: corruption*				
Decile 2–9 of WGI distribution	0.024	*(0.024 - 0.024)*		
Top decile	0.018	*(0.017 - 0.018)*		
				
*History: British Empire*				
Not former British territory			0.017	*(0.017 - 0.017)*
Former British territory			0.037	*(0.037 - 0.038)*
				
*History: No. of E and W residents born in country*				
Less than 50k				
50k-100k				
More than 100k				
				
*History: official / common spoken languages*				
Doesn't include English				
Includes English				
				
*Government: DFID priority for official aid*				
Not focus country				
Focus country				
				
*Geographical scope of charity* (charity-level)				
One country	0.005	*(0.005-0.005)*	0.005	*(0.005-0.005)*
2-9 countries	0.019	*(0.019-0.019)*	0.019	*(0.019-0.019)*
10 countries +	0.146	*(0.144-0.147)*	0.146	*(0.144-0.147)*
				
*N*	*3,271,074*		*3,271,074*	

*Notes*: All models also include controls for the logarithm of the country population size (main effect and squared terms). Number of observations = 201 countries × 16,274 charities = 3,271,074. 95% CI in brackets.

*Source*: author's analysis

**TABLE 3 (cont.):** Logistic regression results (Models A5-A6; considering all charities that operate outside the UK): average predicted probability of a charity working in any given overseas country, by covariate characteristics

	A5		A6	

*Region*				
High income				
East Asia & Pacific				
Europe and Central Asia				
Latin America & Caribbean				
Middle East & North Africa				
South Asia				
Sub-Saharan Africa				
				
*Governance: instability*				
Decile 2–9 of WGI distribution				
Top decile				
				
*Governance: corruption*				
Decile 2–9 of WGI distribution				
Top decile				
				
*History: British Empire*				
Not former British territory				
Former British territory				
				
*History: No. of E and W residents born in country*				
Less than 50k	0.018	*(0.018 - 0.018)*		
50k-100k	0.031	*(0.030 - 0.031)*		
More than 100k	0.046	*(0.045 - 0.047)*		
				
*History: official / common spoken languages*				
Doesn't include English			0.017	*(0.017 - 0.017)*
Includes English			0.038	*(0.037 - 0.038)*
				
*Government: DFID priority for official aid*				
Not focus country				
Focus country				
				
*Geographical scope of charity* (charity-level)				
One country	0.005	*(0.005-0.005)*	0.005	*(0.005-0.005)*
2-9 countries	0.019	*(0.019-0.019)*	0.019	*(0.019-0.019)*
10 countries +	0.146	*(0.144-0.147)*	0.146	*(0.144-0.147)*
				
*N*	*3,271,074*		*3,271,074*	

*Notes*: All models also include controls for the logarithm of the country population size (main effect and squared terms). Number of observations = 201 countries × 16,274 charities = 3,271,074. 95% CI in brackets.

*Source*: author's analysis

**TABLE 3 (cont.):** Logistic regression results (Models A7-A8; considering all charities that operate outside the UK): average predicted probability of a charity working in any given overseas country, by covariate characteristics

	A7		A8	

*Region*				
High income			0.027	*(0.026-0.027)*
East Asia & Pacific			0.015	*(0.014-0.015)*
Europe and Central Asia			0.021	*(0.021-0.022)*
Latin America & Caribbean			0.017	*(0.017-0.018)*
Middle East & North Africa			0.019	*(0.018-0.019)*
South Asia			0.023	*(0.023-0.024)*
Sub-Saharan Africa			0.027	*(0.027-0.028)*
				
*Governance: instability*				
Decile 2–9 of WGI distribution			0.0242	*(0.024-0.024)*
Top decile			0.018	*(0.018-0.019)*
				
*Governance: corruption*				
Decile 2–9 of WGI distribution			0.024	*(0.024-0.024)*
Top decile			0.017	*(0.016-0.017)*
				
*History: British Empire*				
Not former British territory			0.021	*(0.021-0.021)*
Former British territory			0.027	*(0.026-0.027)*
				
*History: No. of E and W residents born in country*				
Less than 50k			0.020	*(0.020-0.021)*
50k-100k			0.029	*(0.028-0.029)*
More than 100k			0.033	*(0.032-0.033)*
				
*History: official / common spoken languages*				
Doesn't include English			0.021	*(0.021-0.021)*
Includes English			0.026	*(0.026-0.027)*
				
*Government: DFID priority for official aid*				
Not focus country	0.019	*(0.019 - 0.019)*	0.021	*(0.021-0.021)*
Focus country	0.039	*(0.039 - 0.040)*	0.031	*(0.031-0.032)*
				
*Geographical scope of charity* (charity-level)				
One country	0.005	*(0.005-0.005)*	0.005	*(0.005-0.005)*
2-9 countries	0.019	*(0.019-0.019)*	0.019	*(0.019-0.019)*
10 countries +	0.146	*(0.144-0.147)*	0.146	*(0.144-0.147)*
				
*N*	*3,271,074*		*3,271,074*	

*Notes*: All models also include controls for the logarithm of the country population size (main effect and squared terms). Number of observations = 201 countries × 16,274 charities = 3,271,074. 95% CI in brackets.

*Source*: author's analysis

**TABLE 3 (cont.):** Logistic regression results (Models A9-A10; considering all charities that operate outside the UK): average predicted probability of a charity working in any given overseas country, by covariate characteristics

	A9		A10	
				
*History: British Empire X Geog. scope of charity*				
One country - Not former British territory	0.002	*(0.002-0.002)*		
One country - Former British territory	0.012	*(0.012-0.012)*		
2-9 countries - Not former British territory	0.011	*(0.011-0.011)*		
2-9 countries - Former British territory	0.036	*(0.036-0.037)*		
10 countries - Not former British territory	0.129	*(0.127-0.130)*		
10 countries - Former British territory	0.189	*(0.187-0.191)*		
				
*History: British Empire X Annual income (£)*				
Under £10k - Not former British territory			0.010	*(0.010-0.011)*
Under £10k - Former British territory			0.028	*(0.028-0.029)*
£10k-£100k - Not former British territory			0.012	*(0.012-0.012)*
£10k-£100k - Former British territory			0.031	*(0.030-0.031)*
£100k-£1m - Not former British territory			0.023	*(0.023-0.024)*
£100k-£1m - Former British territory			0.046	*(0.045-0.047)*
£1m -£10m - Not former British territory			0.048	*(0.047-0.049)*
£1m -£10m - Former British territory			0.083	*(0.081-0.085)*
£10m+ - Not former British territory			0.095	*(0.093-0.098)*
£10m+ - Former British territory			0.169	*(0.166-0.172)*
				
*N*	*3,271,074*		*2,566,971*	

*Notes*: All models also include controls for the logarithm of the country population size (main effect and squared terms). Number of observations (A9: 201 countries × 16,274 charities=3,271,074; A10: 201 countries × 12,771 charities with non-missing income=2,566,971). 95% CI in brackets.

*Source*: author's analysis

English and Welsh charities are most likely to work in countries in South Asia (SA) or in Sub-Saharan Africa (SSA) (Model A1). Charities are over twice (RR=c.0.032/c.0.013=2.5) as likely to work in countries in these two regions than in countries in East Asia and the Pacific (EAP), Latin America and the Caribbean (LAC), the Middle East and North Africa (MENA), or Europe and Central Asia (ECA)^11^. Notably, charities are also more likely to work in high-income countries than in countries in these last four regions.

Charities are less likely to work in countries with low levels of governance: compared to other countries, charities are 13 per cent less likely (RR=1-(0.021/0.024)=0.87) to work in countries that are considered the least politically stable (in the top decile of the WGI's instability distribution) (Model A2), and 25 per cent less likely (RR=1-(0.018/0.024)=0.75) to work in countries where corruption is considered to be least under control (in the top decile of WGI's corruption distribution) (Model A3).

Charities are much more likely to work in countries with historical connections to the UK: compared to other countries, they are more than twice as likely to work in a country that was at some stage a territory that formed part of the British Empire (RR=0.037/.017=2.2) (Model A4); more than twice as likely to work in a country where many people have moved after birth to currently reside in England and Wales (probability of 0.046 for more than 100,000 people, compared to 0.018 for less than 50,000; RR=2.6) (Model A5); and more than twice as likely to work where English is an official or common spoken language (RR=0.038/.017=2.2) (Model A6).

Charities are much more likely to work in countries that are priorities for UK government aid (Model A7): compared to other countries, they are more than twice as likely (RR=0.039/0.019=2.1) to work in a country identified as a ‘focus’ country in the 2011 bilateral aid review.

The main results are robust to model specification. As with Models A1–A7, which consider each of our country-level covariates in turn, a model which includes all seven covariates together shows that charities are more likely to work in countries with colonial and linguistic ties to the UK and less likely to work in countries with high levels of instability or corruption (Model A8). Comparing Model A8 with the previous models also points to the inter-relationships between our covariates. For example in Model A8, when we control for other covariates, there is a smaller difference between former British territories and other countries in the probability of charitable operation than in Model A4. This suggests that associations between a country's colonial past and other covariates – including, for example, the use of the English language – help to explain why charities are more likely to work in former British territories.

Does the importance of the covariates vary according to the size of charity? In Model A9 we find evidence for a significant interaction between the importance of colonial ties and the geographical scope of the charity^12^. A charity with a large scope – working in at least 10 countries – is 50 per cent more likely to work in a particular country if it is a former British territory (RR=0.189/0.129=1.5). However, for charities working in just one country, their operation is even more heavily concentrated in former British territories (RR=0.0121/0.0018=6.7). Similarly, while the largest charities in financial terms – with an annual income greater than £10m – are more likely to work in a country if it is a former British territory (RR:1.7), this pattern is particularly pronounced for the smallest charities (RR: 2.7 and 2.5 for charities with an income of less than £10,000 or between £10,000 and £100,000 respectively) (Model A10).

Does the importance of these covariates extend not only to the population of charities in general, but also specifically to established development organisations? We repeated the modelling process, using the same covariates, for a sub-population of charities recognised as working in international development – members of the umbrella body BOND^13^ (Table 4; Models B1–B8). BOND members are on average much larger than the overseas charitable population as a whole: they had a median income in 2012 of £1.1m, and a mean of £5.2m, compared to £23,400 and £970,500 respectively for the population of charities that work internationally. Given these organisations’ focus on poverty, we use the number of people identified as multi-dimensionally poor in each country (Alkire *et al*., 2014), rather than the total population, as a control. Therefore the frame of comparison is also different^14^: we make comparisons not across 201 countries, but across 98 low- and middle-income countries for which data on acute multi-dimensional poverty are available. There are 28,714 observations across the combination of 293 charities and 98 countries. Table 4 presents the results. After controlling for the number of MPI poor, international development charities are around 50 per cent more likely to work in countries in South Asia and Sub-Saharan Africa than in ECA or MENA (Model B1), though regional differences are reduced when we control for other covariates (Model B8). International development charities are, compared to other countries and after controlling for the number of MPI poor, 19 per cent less likely to work in countries considered the least politically stable (RR=0.81) (Model B2); 25 per cent less likely to work in countries where corruption is considered least under control (RR=0.75) (Model B3); twice as likely to work in former British territories (RR=1.95) (Model B4); more likely to work in a country where many people have moved after birth to currently reside in England and Wales (Model B5); more likely to work where English is an official or common spoken language (RR=1.87) (Model B6); and twice as likely to work in the ‘focus’ countries that are a priority for UK official aid (RR=2.08) (Model B7). Importantly, therefore, these results illustrate considerable unevenness in charitable operation between countries according to our covariates – even for this sub-population of large development charities. **TABLE 4.** Logistic regression results (Models B1-B2; considering only charities that are members of UK overseas development umbrella body BOND): average predicted probability of a charity working in any given overseas country, by covariate characteristics

	B1		B2	

*Region*				
High income	-			
East Asia & Pacific	0.086	*(0.077 - 0.096)*		
Europe and Central Asia	0.083	*(0.073 - 0.094)*		
Latin America & Caribbean	0.101	*(0.092 - 0.110)*		
Middle East & North Africa	0.091	*(0.079 - 0.102)*		
South Asia	0.130	*(0.117 - 0.143)*		
Sub-Saharan Africa	0.126	*(0.121 - 0.132)*		
				
*Governance: instability*				
Decile 2–9 of WGI distribution			0.117	*(0.113 - 0.121)*
Top decile			0.095	*(0.089 - 0.102)*
				
*Governance: corruption*				
Decile 2–9 of WGI distribution				
Top decile				
				
*History: British Empire*				
Not former British territory				
Former British territory				
				
*History: No. of E and W residents born in country*				
Less than 50k				
50k-100k				
More than 100k				
				
*History: official / common spoken languages*				
Doesn't include English				
Includes English				
				
*Government: DFID priority for official aid*				
Not focus country				
Focus country				
	-			
*Geographical scope of charity* (charity-level)				
One country	0.010	*(0.007-0.012)*	0.010	*(0.007-0.012)*
2-9 countries	0.045	*(0.041-0.049)*	0.045	*(0.041-0.049)*
10 countries +	0.224	*(0.216-0.231)*	0.224	*(0.216-0.231)*
				
*N*	*28,714*		*28,714*	

*Notes*: All models also include controls for the logarithm of the number of people identified as multidimensionally poor in each country (main effect and squared terms). Number of observations for all models = 98 countries (with information on multidimensional poverty) × 293 charities (BOND members)=28,714. 95% CI in brackets.

*Source*: author's analysis

**TABLE 4 (cont.):** Logistic regression results (Models B3-B4; considering only charities that are members of UK overseas development umbrella body BOND): average predicted probability of a charity working in any given overseas country, by covariate characteristics

	B3		B4	

*Region*				
High income				
East Asia & Pacific				
Europe and Central Asia				
Latin America & Caribbean				
Middle East & North Africa				
South Asia				
Sub-Saharan Africa				
				
*Governance: instability*				
Decile 2–9 of WGI distribution				
Top decile				
				
*Governance: corruption*				
Decile 2–9 of WGI distribution	0.117	*(0.113 - 0.121)*		
Top decile	0.087	*(0.080 - 0.095)*		
				
*History: British Empire*				
Not former British territory			0.088	*(0.084 - 0.092)*
Former British territory			0.170	*(0.163 - 0.178)*
				
*History: No. of E and W residents born in country*				
Less than 50k				
50k-100k				
More than 100k				
				
*History: official / common spoken languages*				
Doesn't include English				
Includes English				
				
*Government: DFID priority for official aid*				
Not focus country				
Focus country				
				
*Geographical scope of charity* (charity-level)				
One country	0.010	*(0.007-0.012)*	0.010	*(0.007-0.012)*
2-9 countries	0.045	*(0.041-0.049)*	0.045	*(0.041-0.049)*
10 countries +	0.224	*(0.216-0.231)*	0.224	*(0.216-0.231)*
				
*N*	*28,714*		*28,714*	

*Notes*: All models also include controls for the logarithm of the number of people identified as multidimensionally poor in each country (main effect and squared terms). Number of observations for all models = 98 countries (with information on multidimensional poverty) × 293 charities (BOND members) = 28,714. 95% CI in brackets.

*Source*: author's analysis

**TABLE 4 (cont.):** Logistic regression results (Models B5-B6; considering only charities that are members of UK overseas development umbrella body BOND): average predicted probability of a charity working in any given overseas country, by covariate characteristics

	B5		B6	

*Region*				
High income				
East Asia & Pacific				
Europe and Central Asia				
Latin America & Caribbean				
Middle East & North Africa				
South Asia				
Sub-Saharan Africa				
				
*Governance: instability*				
Decile 2–9 of WGI distribution				
Top decile				
				
*Governance: corruption*				
Decile 2–9 of WGI distribution				
Top decile				
				
*History: British Empire*				
Not former British territory				
Former British territory				
				
*History: No. of E and W residents born in country*				
Less than 50k	0.099	*(0.095 - 0.103)*		
50k-100k	0.142	*(0.129 - 0.156)*		
More than 100k	0.157	*(0.145 - 0.170)*		
				
*History: official / common spoken languages*				
Doesn't include English			0.087	*(0.084 - 0.091)*
Includes English			0.163	*(0.156 - 0.171)*
				
*Government: DFID priority for official aid*				
Not focus country				
Focus country				
				
*Geographical scope of charity* (charity-level)				
One country	0.010	*(0.007-0.012)*	0.010	*(0.007-0.012)*
2-9 countries	0.045	*(0.041-0.049)*	0.045	*(0.041-0.049)*
10 countries +	0.224	*(0.216-0.231)*	0.224	*(0.216-0.231)*
				
*N*	*28,714*		*28,714*	

*Notes*: All models also include controls for the logarithm of the number of people identified as multidimensionally poor in each country (main effect and squared terms). Number of observations for all models = 98 countries (with information on multidimensional poverty) × 293 charities (BOND members) = 28,714. 95% CI in brackets.

*Source*: author's analysis

**TABLE 4 (cont.):** Logistic regression results (Models B7-B8; considering only charities that are members of UK overseas development umbrella body BOND): average predicted probability of a charity working in any given overseas country, by covariate characteristics

	B7		B8	

*Region*				
High income			-	
East Asia & Pacific			0.115	*(0.103 - 0.127)*
Europe and Central Asia			0.093	*(0.081 - 0.105)*
Latin America & Caribbean			0.121	*(0.111 - 0.131)*
Middle East & North Africa			0.103	*(0.090 - 0.117)*
South Asia			0.106	*(0.094 - 0.118)*
Sub-Saharan Africa			0.115	*(0.109 - 0.120)*
				
*Governance: instability*				
Decile 2–9 of WGI distribution			0.115	*(0.111 - 0.119)*
Top decile			0.100	*(0.092 - 0.109)*
				
*Governance: corruption*				
Decile 2–9 of WGI distribution			0.118	*(0.113 - 0.122)*
Top decile			0.083	*(0.075 - 0.092)*
				
*History: British Empire*				
Not former British territory			0.101	*(0.096 - 0.106)*
Former British territory			0.131	*(0.122 - 0.141)*
				
*History: No. of E and W residents born in country*				
Less than 50k			0.105	*(0.101 - 0.110)*
50k-100k			0.136	*(0.122 - 0.150)*
More than 100k			0.127	*(0.115 - 0.139)*
				
*History: official / common spoken languages*				
Doesn't include English			0.106	*(0.100 - 0.112)*
Includes English			0.120	*(0.112 - 0.129)*
				
*Government: DFID priority for official aid*				
Not focus country	0.083	*(0.079 - 0.087)*	0.089	*(0.084 - 0.094)*
Focus country	0.173	*(0.164 - 0.181)*	0.155	*(0.145 - 0.165)*
				
*Geographical scope of charity* (charity-level)				
One country	0.010	*(0.007-0.012)*	0.010	*(0.007-0.012)*
2-9 countries	0.045	*(0.041-0.049)*	0.045	*(0.041-0.049)*
10 countries +	0.224	*(0.216-0.231)*	0.224	*(0.216-0.231)*
				
*N*	*28,714*		*28,714*	

*Notes*: All models also include controls for the logarithm of the number of people identified as multidimensionally poor in each country (main effect and squared terms). Number of observations for all models = 98 countries (with information on multidimensional poverty) × 293 charities (BOND members) = 28,714. 95% CI in brackets.

*Source*: author's analysis

### Patterns in country of operation: individual countries

The regression results provide a helpful summary of the relationship between charitable operation and the covariates, which is a useful basis for exploring patterns at the level of individual countries. Figures 3a and 3b^15^ present scatterplots of the total number of charities operating in particular countries, by region, within the context of the countries’ population size (log scale). In Figure 3a, countries indicated by triangles are those that used to be British territories; in Figure 3b, countries indicated by triangles are those in the top decile of the WGI corruption distribution. Countries are labelled using ISO codes, which are listed in Table 5. It is instructive to consider countries on the scatterplots that are outliers, in departing from the general tendency for countries with higher populations to have more charities operating there.

**Figure 3a).**
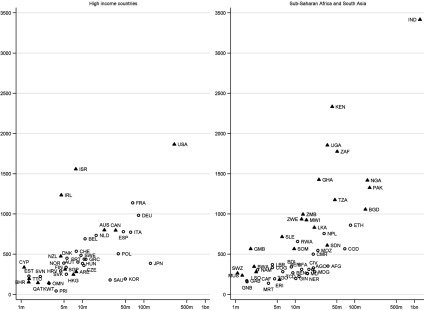
Total number of English and Welsh charities operating in particular countries. *Source:* author's analysis.

*Notes:* Vertical axis: number of charities; horizontal axis: country population (log scale). Triangles show former British territories. For list of country codes see Table 5.

**Figure 3b).**
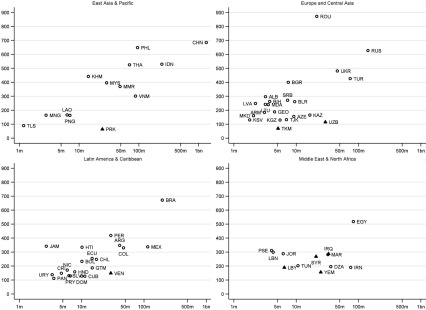
Total number of English and Welsh charities operating in particular countries. *Source:* author's analysis.

*Notes:* Vertical axis: number of charities; horizontal axis: country population (log scale). Triangles show countries that suffer most from corruption (WGI). For country codes see Table 5.

**TABLE 5.** List of ISO country codes, to accompany Figures 3–4

AFG	Afghanistan	LBR	Liberia
AGO	Angola	LBY	Libya
ALB	Albania	LKA	Sri Lanka
ARE	United Arab Emirates	LSO	Lesotho
ARG	Argentina	LTU	Lithuania
ARM	Armenia	LVA	Latvia
AUS	Australia	MAR	Morocco
AUT	Austria	MDA	Moldova, Republic of
AZE	Azerbaijan	MDG	Madagascar
BDI	Burundi	MEX	Mexico
BEL	Belgium	MKD	Macedonia, Republic of
BEN	Benin	MLI	Mali
BFA	Burkina Faso	MMR	Myanmar
BGD	Bangladesh	MNG	Mongolia
BGR	Bulgaria	MOZ	Mozambique
BHR	Bahrain	MRT	Mauritania
BIH	Bosnia and Herzegovina	MUS	Mauritius
BLR	Belarus	MWI	Malawi
BOL	Bolivia, Plurinational State of	MYS	Malaysia
BRA	Brazil	NAM	Namibia
BWA	Botswana	NER	Niger
CAF	Central African Republic	NGA	Nigeria
CAN	Canada	NIC	Nicaragua
CHE	Switzerland	NLD	Netherlands
CHL	Chile	NOR	Norway
CHN	China	NPL	Nepal
CIV	Côte d'Ivoire	NZL	New Zealand
CMR	Cameroon	OMN	Oman
COD	Congo, Dem. Rep.	PAK	Pakistan
COG	Congo	PAN	Panama
COL	Colombia	PER	Peru
CRI	Costa Rica	PHL	Philippines
CUB	Cuba	PNG	Papua New Guinea
CYP	Cyprus	POL	Poland
CZE	Czech Republic	PRI	Puerto Rico
DEU	Germany	PRK	Korea, Dem. People's Rep.
DNK	Denmark	PRT	Portugal
DOM	Dominican Republic	PRY	Paraguay
DZA	Algeria	PSE	Palestine, State of
ECU	Ecuador	QAT	Qatar
EGY	Egypt	ROU	Romania
ERI	Eritrea	RUS	Russian Federation
ESP	Spain	RWA	Rwanda
EST	Estonia	SAU	Saudi Arabia
ETH	Ethiopia	SDN	Sudan
FIN	Finland	SEN	Senegal
FRA	France	SGP	Singapore
GAB	Gabon	SLE	Sierra Leone
GEO	Georgia	SLV	El Salvador
GHA	Ghana	SOM	Somalia
GIN	Guinea	SRB	Serbia
GMB	Gambia	SSD	South Sudan
GNB	Guinea-Bissau	SVK	Slovakia
GRC	Greece	SVN	Slovenia
GTM	Guatemala	SWE	Sweden
HKG	Hong Kong	SWZ	Swaziland
HND	Honduras	SYR	Syrian Arab Republic
HRV	Croatia	TCD	Chad
HTI	Haiti	TGO	Togo
HUN	Hungary	THA	Thailand
IDN	Indonesia	TJK	Tajikistan
IND	India	TKM	Turkmenistan
IRL	Ireland	TLS	Timor-Leste
IRN	Iran, Islamic Republic of	TTO	Trinidad and Tobago
IRQ	Iraq	TUN	Tunisia
ISR	Israel	TUR	Turkey
ITA	Italy	TZA	Tanzania, United Republic of
JAM	Jamaica	UGA	Uganda
JOR	Jordan	UKR	Ukraine
JPN	Japan	URY	Uruguay
KAZ	Kazakhstan	USA	United States
KEN	Kenya	UZB	Uzbekistan
KGZ	Kyrgyzstan	VEN	Venezuela, Bol. Rep.
KHM	Cambodia	VNM	Viet Nam
KOR	Korea, Republic of	YEM	Yemen
KSV	Kosovo	ZAF	South Africa
KWT	Kuwait	ZMB	Zambia
LAO	Lao People's Dem. Rep.	ZWE	Zimbabwe
LBN	Lebanon		

*Note:* This lists the 157 countries with a population of at least 1 million that are displayed in Figures 3–4, not the full population of 201 countries used in the regression models.

Within the group of high-income countries, Israel (ISR) is particularly distinctive as a country where a high number of charities work given its population size (Figure 3a), reflecting the country's religious and ethnic significance. The distinctively high number of charities in Ireland (IRL) reflects the country's close historical links to the UK. Countries that are outside of Europe and not former British territories, like Japan (JPN), South Korea (KOR) and Saudi Arabia (SAU), have a distinctively low number of charities given their population size^16^.

Sub-Saharan Africa and South Asia are of particular interest since charities have the highest propensity to operate in these regions. India, with a very large population and colonial links, has the highest number of charities of any country (Figure 3a). More generally, holding population size constant, countries that used to be British territories (indicated by triangles) tend to have higher numbers of charities. For example, there are 3–4 times more charities working in each of the former British territories of Zimbabwe (ZWE), Zambia (ZMB) and Malawi (MWI) than in countries of comparable size without these historical links: Niger (NER), Mali (MLI), Cote d'Ivoire (CIV), Burkina Faso (BFA), Senegal (SEN), Chad (TCD), Benin (BEN) and Guinea (GIN). Similar differences can be seen for other countries of comparable size but which differ according to whether they are a former British territory: Sierra Leone (SLE) vs. Burundi (BDI) and Togo (TGO); Ghana (GHA) vs. Mozambique (MOZ), Angola (AGO) and Madagascar (MDG). However, it is also clear that there is considerable variation in charitable operation even among these countries with colonial ties: there are particularly high numbers of charities working in Kenya (KEN), Uganda (UGA) and South Africa (ZAF) – more than in other former British territories with comparable or larger populations, including Tanzania (TZA) and Nigeria (NGA) and, in South Asia, Pakistan (PAK) and Bangladesh (BGD). Meanwhile, given their population size, there are relatively low numbers of charities in many of the countries that are in the top decile of WGI's instability distribution – indicating those considered least politically stable / most at risk of politically-motivated violence and terrorism – including Ethiopia (ETH), Democratic Republic of Congo (COD), Sudan (SDN), Afghanistan (AFG), NER, and South Sudan (SSD).

In Europe and Central Asia, a distinctively high number of charities work in Romania (ROU) (Figure 3b; note the different vertical axis scale compared to Figure 3a). Generally, for a given population size, more charities operate in European countries (e.g. ALB, BGR, BLR, LVA, MDA, SRB) than in the Caucasus (ARM, AZE, GEO) and Central Asia (KAZ, KGZ, TKM, TJK, UZB). In the Latin American and Caribbean region, given its population size, Jamaica (JAM) has a high number of charities, reflecting its historical connections to the UK. In the Middle East, more charities work in the Palestinian territories (PSE), Lebanon (LBN) and Jordan (JOR) than, for example, populous but relatively closed Iran. Given their population size, North Korea (PRK), Turkmenistan (TKM), Uzbekistan (UZB), and Venezuela (VEN) all stand out in their respective regions as countries where few charities operate. Notably these countries (indicated by triangles) are all in the top decile of the WGI corruption distribution.

**Figure 4.**
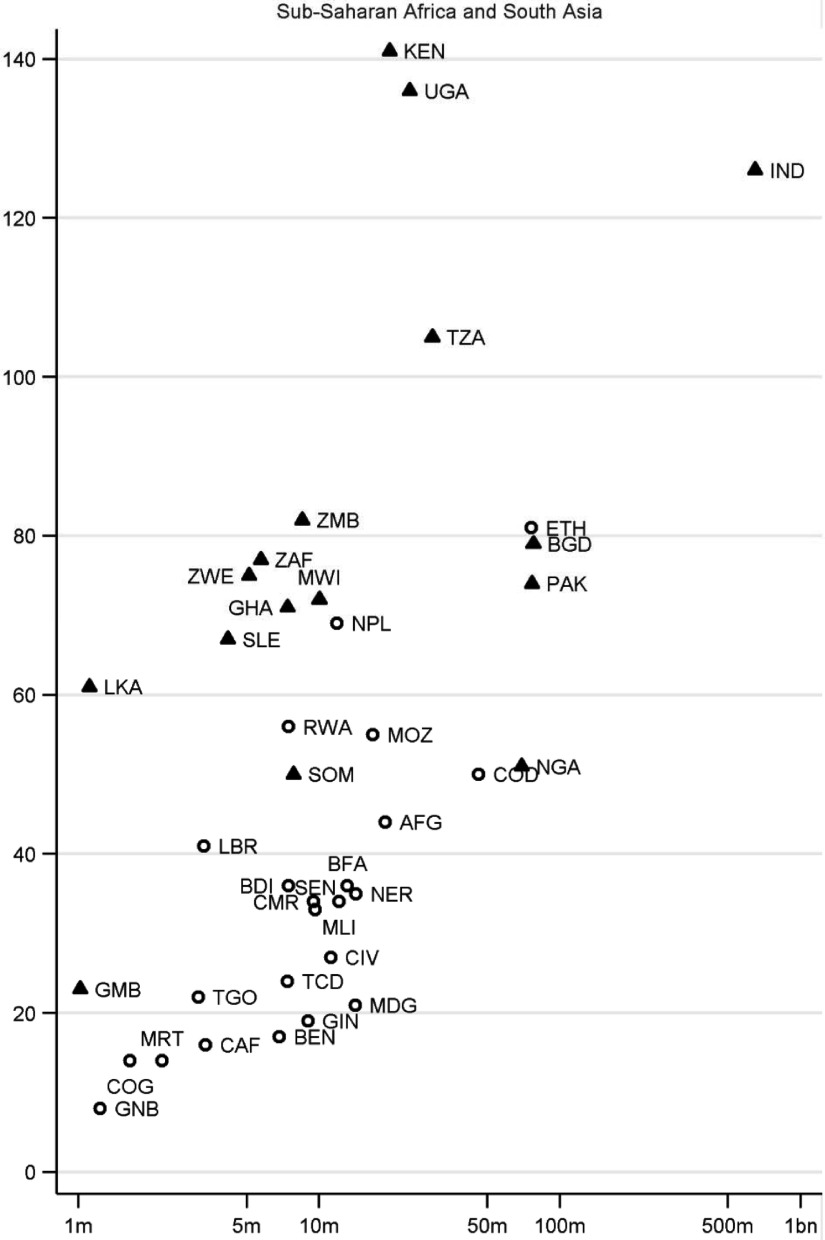
Number of members of UK overseas development umbrella body BOND, operating in particular countries. *Source:* author's analysis.

*Notes:* Vertical axis: number of charities; horizontal axis: number of multidimensionally poor (log scale). Triangles show former British territories. For list of country codes see Table 5.

What are the patterns by country for the specific sub-population of BOND members involved in international development? Figure 4 provides a scatterplot for these charities, with the horizontal axis representing not the total population but the number of people who are multi-dimensionally poor in each country^17^. Only two regions are included – SA and SSA – which are collectively home to 80 per cent of the world's multidimensional poor. Interestingly, the country pattern in operation for the 293 BOND members – which are on average much larger than the other overseas charities – is generally similar to that for the 16,274 charities as a whole. Thus, former British territories tend to have a much higher number of international development charities than other countries given the number of multi-dimensionally poor: for example, ZWE, ZMB, MWI, SLE have between c.2 and 4 times more BOND members operating than do NER, BFA, MLI, SEN, BDI, CIV, TCD, MDG, GIN, and TGO. As before, there is variation even among the countries with colonial ties, with KEN and UGA particularly distinctive in the high number of BOND members that operate there.

The results in Figures 3a, 3b and 4 make it possible to compare current priorities for UK official aid, in terms of countries that were chosen as one of 27 ‘focus countries’ in the DFID bilateral review, with country patterns in the operation of charities registered in England and Wales. Many of the focus countries are also countries where a high number of charities operate (for example, KEN, UGA, and TZA). Nevertheless there are some focus countries, including some chosen as part of the UK government's commitment to support fragile and conflict-afflicted states, where the number of charities operating is relatively low (for example: Yemen (YEM), PSE in MENA; COD, Liberia (LBR) in SSA; AFG in SA; Kyrgyzstan (KGZ), Tajikistan (TJK) in ECA). It is interesting to note countries with a high development need but which are not DFID focus countries, and where a relatively low number of charities operate (for example, AGO; BEN; BDI; BFA; CIV; GIN; MDG; MLI; NER; SEN; TCD). In these countries the lack of historical UK ties may help to explain not only the low level of charitable operation but also DFID's decision not to prioritise countries where, compared to other bilateral programmes or compared to multilateral aid, there was felt to be a lack of UK comparative advantage in delivering aid (DFID, 2011).

## Discussion

This paper provides new information about trends in the number, and patterns in the geographical reach, of English and Welsh charities operating internationally. It adopts a distinctive empirical approach. In examining the population of international voluntary organisations that collectively work across every country globally, we move away from a binary focus on either ‘developing’ or ‘developed’ country contexts (Lewis, 2014). In examining the full size distribution of charities, including small ‘grassroots’ charities as well as large professionalised organisations, we gain insight into the international connections provided by ‘non-elite’ as well as ‘elite’ actors (see Yeates and Holden, 2009). The results therefore provide a fresh empirical perspective on international charitable activity: while it is the large charities working in overseas development in aid-recipient contexts that are household names, there is perhaps less public awareness of the extent of international activity that takes place through small-scale ‘grassroots’ registered charities, and of the extent of activity in ‘developed’, as well as ‘developing’, country contexts.

### Trends over time

This paper documents a sizeable increase in the number of voluntary organisations working internationally. The results are consistent with the possibilities for international collaboration provided by accessible methods of travel and communication. Importantly, recognising the significance of these technologies does not equate to ‘assigning them as a determining role’ in international processes given their mediation by a variety of factors (Yeates, 2008: 4). It should also be emphasised that international civil society is not new, and has been facilitated by technological improvements for hundreds of years (Davies, 2013). Instead the focus is on the continuing processes of integration that ‘bring together’ or ‘enmesh’ the lives of ‘distant people and places around the world’ (Yeates, 2008: 3; Held *et al.*, 1999).

Importantly, while there has been a sizeable increase in the number of large charities that work overseas since the mid-1990s, there has been a particularly significant increase in the number of small organisations. These results are consistent with the growing importance of small-scale ‘grassroots’ international voluntary organisations (Schnable, 2014) or international citizen ‘private initiatives’ (Kinsbergen and Schulpen, 2013). These tend to be small organisations, founded by non-development specialists, run by volunteers and funded by individuals, focused on the direct provision of goods or services to individuals and communities overseas (Schnable, 2014; Kinsbergen and Schulpen, 2009; 2013; Kinsbergen *et al.*, 2013). These small-scale organisations are little discussed in academic and policy circles. Recent policy interest in private actors in development has focused instead on the role of large-scale foundations and high-net-worth donors (International Development Committee, 2012; see Bishop and Green, 2008, Hénon, 2014). World culture theorists emphasise the networks of large-scale NGOs rather than the networks of ‘small-scale altruists’ (see Hannan, 2012). More generally, empirical research on small international voluntary organisations has been limited by a lack of available data (Develtere and De Bruyn, 2009). Therefore this paper, which for the first time provides insight into the sizeable number of small English and Welsh charities operating overseas, provides important empirical support for Schnable's (forthcoming) call for ‘conversations about globalisation to be voluntarised’ and for ‘conversations about [voluntary organisations] and voluntarism to be globalised’ – ‘to take into account the small scale voluntary groups that are linking distant communities’.

### Patterns in country of operation

The paper describes, for the first time, patterns in the countries of operation of English and Welsh charities working overseas. It illustrates considerable unevenness in charitable operation, even after controlling for total population size or the total number of people in poverty. This aligns with the emerging literature on the aid allocation of international development NGOs which, while showing that development need is indeed a predictor of aid allocation, also illustrates that international NGOs are more likely to operate in countries with shared colonial ties, are more likely to operate in countries that are prioritised by the respective official donors, and are less likely to engage in countries with poor governance (Nunnenkamp *et al.*, 2009; Koch, 2009; Dreher *et al*., 2010; Dreher *et al*., 2012; Büthe *et al.*, 2012). However, these existing studies focus on a small number of large NGOs involved in development – for example, 40–50 of the largest international NGOs in Germany or in the US – and only compare the operation of organisations across aid-recipient countries. Therefore, while these studies successfully capture the bulk of international financial flows, they miss much of the voluntary activity that links people and places internationally. This paper shows that there are more than 10,000 charities in England and Wales with annual incomes of under £100,000 that operate overseas, involving around 54,000 trustees in their operation. Thus this paper makes a distinct and complementary contribution to the body of existing research. For the first time, we illustrate considerable unevenness in patterns of operation across ‘developed’, as well as ‘developing’ country contexts, and for charitable organisations in general – encompassing not just large development NGOs but also large numbers of small ‘grassroots’ voluntary organisations.

These results have theoretical implications. Economic demand-side factors explain the existence of voluntary organisations as a response to the need for goods and services that are undersupplied by the market and the state (for example, Weisbrod, 1975), and as reflecting the need for a nonprofit-distributing constraint as an assurance to donors ‘purchasing’ services for third parties with whom they have little contact (Hansmann, 1996). However the supply of resources, and the institutional context, are also considered important to an understanding of voluntary activity (see DiMaggio and Anheier, 1990). Indeed, differences in the supply of human and financial resources provide a strong theoretical basis for expecting geographical unevenness in voluntary activity (see James, 1987; Salamon, 1987). However, while the potential for unevenness in voluntary activity has been a prominent theme in research for many years, thus far there has been relatively little empirical work to complement and test existing theory. Significantly, the few existing empirical studies have focused on examining geographical variations in the activity of domestic voluntary organisations within industrialised country contexts (for example, Bielefeld and Murdoch, 2004). In contrast there has been a lack of empirical research, outside the specific focus on large development organisations within the aid allocation literature, into the geography of operation of international voluntary organisations. Therefore this paper's distinctive empirical evidence, showing the considerable variation in charitable operation across countries even after controlling for population size and poverty, serves to illustrate the importance of supply-side theories, as well as demand-side factors, to an understanding of international voluntary activity. It is able to show unevenness in the international activity of English and Welsh charities that is consistent with differences in the institutional environment – with fewer charities operating in countries with poor governance – and consistent with differences in the supply of entrepreneurs along colonial lines – with fewer charities operating in countries without historic and linguistic ties with the UK.

These patterns suggest that the ‘philanthropic particularism’ (Salamon, 1987) of English and Welsh charities operating internationally – the clear tendency to focus on certain countries rather than others – cannot be understood independently from historically embedded social and institutional networks (see Bebbington and Kothari, 2006). This insight applies to the population of international charities as a whole, but the results also illustrate its particular relevance for smaller organisations: the c.9,000 charities that work in just one country overseas, which have a median annual income of £17,000, are 6.7 times more likely to work in former British territories than in other countries. This is consistent with descriptive accounts that point to the importance of social networks to the emergence of these ‘grassroots’ international voluntary organisations. Thus Schnable (2014) and Kinsbergen and Schulpen (2013) argue that many emerge from direct personal relationships that are formed through travel to overseas countries. The corollary is that, while they may emerge out of ‘coincidental’ encounters (Kinsbergen and Schulpen, 2013: 57), the geographical distribution of these organisations may be also socially structured by the strength of existing ties between countries, and by path-dependent processes as the social networks created through these grassroots organisations reinforce themselves (Koch, 2009).

## Conclusion

This paper has examined the international activity of registered charities in England and Wales. The paper's results – showing a sizeable increase in the number of charities working internationally, and considerable unevenness in their countries of operation – represent new empirical evidence in an area of research which has been relatively neglected within social policy. However we anticipate that these results will be of considerable public, and not only scholarly, interest. Members of the public, given the high profile of international voluntary organisations and the significant voluntary donations to international causes (see Atkinson *et al.*, 2012; Micklewright and Schnepf, 2009), may be interested to learn more about the nature of international charitable activity. The hundreds of established development charities that are members of BOND, together with the wider population of 16,500 voluntary organisations and the associated 83,000 trustees, will benefit from the information needed to place their international activity within a wider context. In addition the files prepared during the paper's analysis, including a file for each of the 201 countries considered with details of every registered charity working there, are being made available to users through the UK Data Service and should be of considerable practical use (see part 2 of online supplementary material). They will be of interest, for example, to grant-making bodies looking to fund organisations working in a particular country. They will also provide a basis for information-sharing where, as Kinsbergen and Schulpen (2013) point out, there is often limited knowledge about other organisations working in the same country context. Indeed the author recently liaised with BOND, who are involved with information-sharing and coordination activities for UK NGOs working in Ebola-affected countries, to provide them with a database on the organisations with experience of working in these countries. The paper therefore serves to illustrate the potential of this strand of research. Further research, making use of newly available data on voluntary organisations, promises not only to further enhance our understanding of international voluntary activity but also to inform policy and practice.

## Supplementary material

For supplementary material accompanying this paper visit http://dx.doi.org/10.1017/S0047279416000076. click here to view supplementary material

## Notes

1 The computerisation of the Charity Commission Register took place in the early 1990s, so information is not available before 1995.

2 ‘Common’ means spoken by at least 9 per cent of the population.

3 While 16,502 charities indicate that they operate overseas, when describing countries of operation we do not consider 46 minor territories listed in the Charity Commission dataset. In addition, 165 charities that ticked every single country worldwide – including Antarctica and each of the minor territories – were excluded.

4 This represents the sample proportion of observations coded 1 for our outcome variable, which sums to a total of 75,944 (0.023*3,271,074) across the 3,271,074 observations.

5 We take the logarithm of the population size, then include both its main effect and squared terms, having used likelihood ratio tests to compare different models with different functional forms for population.

6 Analysis of allocation is precluded by a lack of data on expenditure by country.

7 This total includes charities that operate both within England and Wales and internationally.

8 Here we do not consider Scotland and Northern Ireland as ‘overseas’.

9 These figures relate to headline income in financial years that ended in 2012, the most recent data available. Percentages do not include the 2,819 charities with missing information for 2012 income.

10 Income data over the period are converted to 2012 prices using the Retail Price Index RPIX.

11 This regional classification treats high-income countries as a separate category.

12 We tested for the significance of this interaction by performing a likelihood ratio test comparing two nested models (Models A9 and A4).

13 As an umbrella body for organisations working in international development, BOND promotes and supports the interests of its members, seeking to influence policy makers and build organisations’ effectiveness. We obtained a list of BOND members and matched this to the data on charities.

14 Thus it is unhelpful to compare the size of the relative risks with those from the earlier models in Table 3.

15 In Figures 3–4 we do not include countries with a population of less than one million.

16 The low number of charities in these three countries may also reflect these countries’ affluence.

17 The figure doesn't include the following countries with population sizes bigger than 1m but without MPI information: Sudan (70 BOND members); Angola (27); South Sudan (21); Botswana (18); Eritrea (8); Mauritius (6).

## References

[ref001] Agg C. (2006), Trends in Government Support for Non Governmental Organisations, Geneva: UNRISD.

[ref002] Alkire S., Conconi A. and Seth S. (2014), Multidimensional Poverty Index 2014: Brief Methodological Note and Results, Oxford: OPHI.

[ref003] Atkinson A.B., Backus P.G., Micklewright J., Pharoah C. and Schnepf S.V. (2012), ‘Charitable Giving for Overseas Development: UK Trends over a Quarter Century’, *Journal of the Royal Statistical Society: Series A*, 167–190.

[ref004] Bebbington A. and Kothari U. (2006), ‘Transnational Development Networks’, Environment and Planning A, 38: 5, 849–866.

[ref005] Bielefeld W. and Murdoch J. (2004), ‘The Locations of Nonprofit Organisations and Their for-Profit Counterparts: An Exploratory Analysis’, Nonprofit and Voluntary Sector Quarterly, 33: 2, 221–246.

[ref006] Bishop M. and Green M. (2008), Philanthrocapitalism: How the Rich Can Save the World, Bloomsbury Press: London.

[ref007] Büthe T., Major S. and de Mello e Souza A. (2012), ‘The Politics of Private Foreign Aid: Humanitarian Principles, Economic Development Objectives, and Organisational Interests in NGO Private Aid Allocation’, International Organisation, 66: 04, 571–607.

[ref008] Christian Aid (2011), Christian Aid Annual Report 2010/2011, London: Christian Aid.

[ref009] Davies T. (2013), NGOs: A New History of Transnational Civil Society, London: Hurst & Company.

[ref010] Department for International Development (DFID) (2011), Bilateral Aid Review: Technical Report, London: DFID.

[ref011] Develtere P. and De Bruyn T. (2009), ‘The Emergence of a Fourth Pillar in Development Aid’, Development in practice, 19: 7, 912–922.

[ref012] DiMaggio P. J. and Anheier H. K. (1990), ‘The Sociology of Nonprofit Organisations and Sectors’, *Annual Review of Sociology*, 137–159.

[ref013] Dreher A., Mölders F. and Nunnenkamp P. (2010), ‘Aid Delivery through Non-Governmental Organisations: Does the Aid Channel Matter for the Targeting of Swedish Aid?’, The World Economy, 33: 2, 147–176.

[ref014] Dreher A., Nunnenkamp P., Thiel S. and Thiele R. (2012), ‘Aid Allocation by German NGOs: Does the Degree of Official Financing Matter?’, The World Economy, 35: 11, 1448–1472.

[ref015] Gough I. and Wood G. (2004), Insecurity and Welfare Regimes in Asia, Africa and Latin America: Social Policy in Development Contexts, Cambridge: Cambridge University Press.

[ref016] Hannan T. (2012), ‘World Culture at the World's Periphery: The Role of Small-Scale Transnational Altruistic Networks in the Diffusion of World Culture’, *Am. Sociol. Assoc. Annu. Meet., Denver, CO, Aug. 17–20*.

[ref017] Hansmann H. (1996), The Ownership of Enterprise, London: Harvard University Press.

[ref018] Held D., McGrew A.G., Goldblatt D. and Perraton J. (1999), Global Transformations: Politics, Economics and Culture, Stanford: Stanford University Press.

[ref019] Hénon S. (2014), Measuring Private Development Assistance: Emerging Trends and Challenges, Bristol: Development Initiatives.

[ref020] International Development Committee (2012), Private Foundations: Thirteenth Report of Session 2010–2012, London: The Stationery Office Limited.

[ref021] James E. (1987), 'The Nonprofit Sector in Comparative Perspective, The Nonprofit Sector: A Research Handbook, New Haven, Connecticut: Yale University Press.

[ref022] Kaufmann D., Kraay A. and Mastruzzi M. (2011), 'The Worldwide Governance Indicators: Methodology and Analytical Issues', Hague Journal on the Rule of Law, 3: 02, 220–246.

[ref023] Kendall J. and Knapp M. (1993), ‘Defining the Nonprofit Sector: The United Kingdom’, *Working Papers of the John Hopkins Comparative Nonprofit Sector Project*.

[ref024] Kinsbergen S. and Schulpen L. (2009), ‘Taking Stock of PIs: The What, Why and How of Private Initiatives in Development’, in P. Hoebink (ed.), The Netherlands Yearbook on International Cooperation, Assen, The Netherlands: Royal Van Gorcum.

[ref025] Kinsbergen S. and Schulpen L. (2013), ‘From Tourist to Development Worker. Private Development Initiatives in the Netherlands’, Mondes en développement: 1, 49–62.

[ref026] Kinsbergen S., Tolsma J. and Ruiter S. (2013), ‘Bringing the Beneficiary Closer: Explanations for Volunteering Time in Dutch Private Development Initiatives’, Nonprofit and Voluntary Sector Quarterly, 42: 1, 59–83.

[ref027] Koch D.-J. (2009), Aid from International NGOs: Blind Spots on the Aid Allocation Map, Abingdon: Routledge.

[ref028] Lewis D. (2013), ‘Building the Welfare Mix or Sidelining the State? Non-Governmental Organisations in Developing Countries as Social Policy Actors’, in R. Surender and R. Walker (eds.), Social Policy in a Developing World, Cheltenham: Edward Elgar.

[ref029] Lewis D. (2014), ‘Heading South: Time to Abandon the ‘Parallel Worlds’ of International Non-Governmental Organisation (NGO) and Domestic Third Sector Scholarship?’, *Voluntas: International Journal of Voluntary and Nonprofit Organisations*, 1–19.

[ref030] Mayer T. and Zignago S. (2011), *Notes on Cepii's Distances Measures: The Geodist Database*: CEPII Document de Travail No 2011–25.

[ref031] Micklewright J. and Schnepf S.V. (2009), ‘Who Gives Charitable Donations for Overseas Development?’, Journal of Social Policy, 38: 02, 317–341.

[ref032] Nunnenkamp P., Weingarth J. and Weisser J. (2009), ‘Is NGO Aid Not So Different after All? Comparing the Allocation of Swiss Aid by Private and Official Donors’, European Journal of Political Economy, 25: 4, 422–438.

[ref033] Office for National Statistics (ONS) (2013), Qs213ew: 2011 Census: Country of Birth (Expanded), Regions in England and Wales, Titchfield: Office for National Statistics.

[ref034] Salamon L. (1987), ‘Of Market Failure, Voluntary Failure, and Third-Party Government: Toward a Theory of Government-Nonprofit Relations in the Modern Welfare State’, Nonprofit and Voluntary Sector Quarterly, 16: 1–2, 29–49.

[ref035] Salamon L. and Anheier H. (1992), ‘In Search of the Non-Profit Sector. I: The Question of Definitions’, Voluntas: International Journal of Voluntary and Nonprofit Organisations, 3: 2, 125–151.

[ref036] Schnable A. (2014), ‘Exporting Bootstraps: Aid Narratives and Grassroots NGOs’, Center for the Study of Social Organisation Working Paper 8.

[ref037] Schnable A. (Forthcoming), ‘New American Relief and Development Organisations: Voluntarizing Global Aid’, *Social Problems*.

[ref038] Solimano A. (2005), ‘Remittances by Emigrants: Issues and Evidence’, in A. Atkinson (ed.), New Sources of Development Finance, Oxford: Oxford University Press.

[ref039] Surender R. and Walker R. (2013), Social Policy in a Developing World, Cheltenham: Edward Elgar.

[ref040] Vakil A.C. (1997), ‘Confronting the Classification Problem: Toward a Taxonomy of NGOs’, World Development, 25: 12, 2057–2070.

[ref041] Van Hear N., Pieke F. and Vertovec S. (2004), The Contribution of UK-Based Diasporas to Development and Poverty Reduction, Oxford: COMPAS, University of Oxford.

[ref042] Watkins S.C., Swidler A. and Hannan T. (2012), ‘Outsourcing Social Transformation: Development NGOs as Organisations’, Annual Review of Sociology, 38, 285–315.

[ref043] Weisbrod B.A. (1975), 'Toward a Theory of the Voluntary Non-Profit Sector, Altrusim, Morality and Economic Theory, New York: Russell Sage.

[ref044] World Bank (2012), *Country and Lending Groups. Available from* Http://Data.Worldbank.Org/About/Country-and-Lending-Groups.

[ref045] World Bank (2013), *Population Statistics by Country. Available from* Http://Databank.Worldbank.Org/Data/Databases/Population-Dynamics.

[ref046] Yeates N. (2008), Understanding Global Social Policy, Bristol: Policy Press.

[ref047] Yeates N. and Holden C. (2009), The Global Social Policy Reader, Bristol: Policy Press.

