# Supplementary material for: International Charitable Connections: the Growth in Number, and the Countries of Operation, of English and Welsh Charities Working Overseas
Source: J Soc Policy. 2016 Jul;45(3):453–86. doi: 10.1017/S0047279416000076 (PMC4890346; doi:10.1017/S0047279416000076)
Supplement: Supplementary file 1 [file S0047279416000076sup001.docx]

**Online supplementary material:**

**guide to data on English and Welsh charities working internationally**

___________________________________________________________________________

This guide provides supplementary information about the data which formed the basis for the analysis in the paper ‘International charitable connections: the growth in number, and the countries of operation, of English and Welsh charities working overseas’.

The guide provides:

1) Further information about the data used in the paper

2) Links to new data resources produced during the analysis. This includes 201 files, with ISO codes, providing information on every charity operating in each of the 201 countries considered.

**1. Further data information**

In this section we consider four questions related to our data: (1) What are charities and which organisations are included in our data? (2) What is distinctive about the Charity Commission data? (3) What information on overseas operation is provided, and what is the quality of this information? (4) How was country-level covariate data linked to the data on charitable operation?

*1.1 What are charities and which organisations are included in our data?*

According to charitable law, charities are voluntary organisations that work for public benefit and have charitable purposes. Charitable purposes include the four heads of charity: the relief of poverty; the advancement of education; the advancement of religion; and other purposes beneficial to the community.

The data that form the basis for the analysis in this paper are from the Register of the Charity Commission for England and Wales. Note:

- Only information on registered charities is provided. Non-charitable voluntary organisations, and charities that are ‘excepted’ or ‘exempted’ from registration, are not included.
- Only charities registered in England and Wales with the Charity Commission are included. Charities registered in Scotland or Northern Ireland, and not with the Charity Commission in England and Wales, are not included.

*1.2 What is distinctive about the Charity Commission data?*

The data provide the basis for the paper’s distinctiveness. First, unlike in many countries where registration is not compulsory and which have a less comprehensive listing of organisations (United Nations, 2011; Hénon, 2014), the CC’s system of registration and annual reporting for charities is well established. Second, unusually, the data contain information on each country in which every charity operates internationally: typically, even where established regulators do exist in other countries, this information is not collected. Third, this country-level information is available for the population of registered charities, not only for the largest organisations. Together these aspects make the CC data unique. In the United States context, for example, information about non-profits’ activity outside the US is recorded at the level of world regions, precluding country-level analysis, and only for non-profits above the threshold of either $500,000 in assets or $200,000 in receipts. Since smaller organisations also tend to be unaffiliated with umbrella organisations, this means that there is no central source of information on US-based ‘grassroots’ international non-profits (Schnable, 2014). This mirrors the situation in other countries, which lack central administrative systems for collecting information on small international voluntary organisations (Pollet *et al*., 2014). The CC data therefore provide a unique opportunity to provide empirical evidence about the full size distribution of registered charitable organisations, including not only large but also ‘grassroots’ organisations, that operate internationally.

*1.3 What information on overseas operation is provided? What is the quality of this information?*

In the annual return each charity must indicate their ‘areas of operation’, including each country outside England and Wales in which the charity operates. The ‘area of operation’ is ‘the geographical area where the charity does its work or provides its benefit.’

There are certain factors which promote confidence in the overall quality of the information on charities’ country of operation:

- There are no implicit incentives for charities to understate or overstate the number of countries in which they operate.
- It is straightforward for charities to update the information annually. The Charity Commission annual return provides a facility through which charities can remove countries from the previous year’s information, and add further countries from a standardised list.

We performed certain data quality checks:

- We examined the distribution of charities according to their geographical scope – in terms of the reported number of countries of operation. We noted 165 charities that implausibly indicated operation in every single country worldwide – including Antarctica and each of the minor territories. These were excluded from the geographical analysis. Note that most of these 165 charities (85 charities, or 52 per cent) were also missing information on 2012 income. The remaining 80 of the 165 charities were quite equally distributed in terms of the proportion within each of the income categories: 30 charities under £10k (0.64 per cent of charities within this size category); 27 charities between £10k and £100k (0.50 per cent); 13 charities between £100k and £1m (0.50 per cent); 9 charities between £1m and £10m (1.08 per cent) and 1 charity above £10m (0.51 per cent).
- We examined the distribution of overseas charities according to their 2012 income. While around a third of overseas charities have an annual income under £10k (4,626 of the 13,683 charities with non-missing income=34 per cent), 1,690 (12 per cent) have an income under £1k and 778 (5 per cent) report an income of 0 for that year. However we do not exclude charities with negligible or zero income in 2012 from the analysis. Note that charities that operate overseas are not distinctive in having a tail of organisations with low incomes: indeed, compared to the population of c.160,000 charities as a whole that are registered with the Charity Commission, a lower proportion of overseas charities have very small incomes. Note too that the income of the very smallest charities is often irregular: around half of charities with zero income in a particular year receive income in the preceding or subsequent year.
- We examined the bivariate distribution of charities’ income and reported geographical scope. Table A1 below is a more detailed version of Table 2 in the paper, with a finer classification of income. The overall relationship is as would be expected: overall, charities with a higher income tend to have a wider scope. However, the cells towards the top right of the table – where charities with negligible income report a wide geographical scope – represent strange cases. Some of these strange cases, where charities indicated operation in every single country worldwide, have already been flagged as implausible and are excluded from the geographical analysis (see above). We investigated the remaining cases empirically using the other information in our data. Our estimate is that in around half of these cases there are particular reasons for a zero or negligible income in 2012. First, the organisations only registered in 2011 or 2012, such that the work of the charity may still be being set up. Second, many charities had consistently much higher income for a number of previous years, such that the negligible income in 2012 may be consistent with the winding down of charitable operations: since there is little incentive for a charity to remove itself from the Register, the dissolution of a charity is often an extended process. Therefore we estimate that, in around half of the cases where charities with negligible income report a wide geographical scope, this is reflective of a particular stage in the organisation’s life cycle.

TABLE A1. Number of charities working internationally, by annual income (£; detailed breakdown) and geographical scope

|  | Geographical scope (number of countries) | | |  |
| --- | --- | --- | --- | --- |
|  | One | 2–9 | 10+ | Total |
| £0 | 465 | 258 | 55 | 778 |
|  | *(60)* | *(33)* | *(7)* |  |
| £1–£1k | 581 | 279 | 52 | 912 |
|  | *(64)* | *(31)* | *(6)* |  |
| £1k–£10k | 1,880 | 893 | 163 | 2,936 |
|  | *(64)* | *(30)* | *(6)* |  |
| £10k–£100k | 3,137 | 1,889 | 353 | 5,379 |
|  | *(58)* | *(35)* | *(7)* |  |
| £100k–£1m | 1,190 | 1,063 | 380 | 2,633 |
|  | *(45)* | *(40)* | *(14)* |  |
| £1m –£10m | 245 | 340 | 263 | 848 |
|  | *(29)* | *(40)* | *(32)* |  |
| £10m+ | 33 | 63 | 101 | 197 |
|  | *(17)* | *(32)* | *(51)* |  |
| Missing income | 1,519 | 963 | 337 | 2,819 |
|  | *(54)* | *(34)* | *(12)* |  |
| Total | 9,050 | 5,748 | 1,704 | 16,502 |
|  | *(55)* | *(35)* | *(10)* |  |

*Notes:* Row percentages in brackets. Annual income in 2012.

However, even after taking life cycle into account, there remain some cases where charities with a limited income report an extended geographical scope. This may reflect two interrelated measurement issues, which are particularly relevant for the smaller charities:

- The interpretation of operation. The Charity Commission asks charities to report their ‘areas of operation’. However, beyond stipulating that this represents ‘the geographical area where the charity does its work or provides its benefit’, they provide no guidance about what this means in practice. Therefore, it is possible that for some of the smallest charities the area of operation may be reflective of relational aspects of their work which need not entail significant financial engagement.
- How charitable operation is distributed over time. While it is straightforward for charities to update their countries of operation each year, they may not choose to do so – even if their level of operation in particular countries is irregular over time. Indeed, particularly for charities whose operation is based on relationships with particular individuals overseas, they may recognise an ongoing commitment to a particular country despite a relative lack of engagement over the last 12 months.

We considered the implications of these measurement issues for our results. In particular, we note that:

- The number of implausible cases, where charities report operating in 10 or more countries despite very limited income, is small. This number is further reduced when we recognise that a high proportion of these cases seem to relate to the stage in the organisation’s life cycle.
- There is no evidence that these cases are related systematically to the country of operation – such that the geographical analysis in the paper should be unaffected.

Therefore we consider the results in this paper to be robust to these measurement issues. Finally it is worth recognising that, while these measurement issues are relevant methodologically, we also see them as interesting from a substantive perspective. Indeed while the number of small international charitable organisations has increased, they have received little attention from a research perspective. Therefore it would be interesting for future research to consider how the operation of the small international charitable organisations is distributed over time – and the potential for ‘episodic’ engagement given their reliance on volunteers.

*1.4 How was country-level covariate data linked to the data on charitable operation?*

This was a two-stage process. First, country codes from the sources that used their own coding systems – including the Charity Commission, DFID, and ONS – were manually recoded to common codes published by the International Organization for Standardization (ISO). Second, using the ISO codes as a common key, the covariate data were merged into a common file (Figure A1, below).

**Governance**

WGI measures of instability and corruption

*(Kaufman et al., 2011)*

**Region**

Country classification *(World Bank, 2012)*

**Charitable operation**

Country operation of

EW charities

*(Charity Commission)*

Figure A1. Bringing data together, using ISO country codes as a common key

ISO

country

code

**Country of birth**

*No. of E and W residents born in country*

*(ONS, 2013)*

**Former British territory; English as official or spoken language**

*(Mayer and Zignago, 2011)*

**Government priorities**

Country priorities for

UK bilateral aid

*(DFID, 2011)*

**Population size**

*(World Bank, 2013)*

**Poverty**

Number of multidimensionally poor *(Alkire et al., 2014)*

**2. Links to new data resources: information on charities working within each country**

**Files**

The files prepared during the paper’s analysis, providing details of EW charities working in each country worldwide, are being made available to users through the UK Data Service (by searching under the project Grant Number: **ES/K00137X/1).** yet have a link availablee
 (the project Grant Number: a Service (s (in this case, the distribution according to income).

There are two different kinds of files.

1. **201 x Country files**: listing charities operating in each of the 201 countries considered
2. **1 x Collated file:**  Listing all 16,274 charities that work across the 201 countries.

**Variables**

Variables common to all files

| *regno* | Charity Commission registration number |
| --- | --- |
| *income_2012* | Headline income (£) for financial years ending at some point in the 2012 calendar year |
| *reg_year* | Year of registration with the Charity Commission |
| *total_countries* | Total number of countries in which charity operates (not including England and Wales/ Scotland/ Northern Ireland) |
| *BOND member* | 1=BOND member; 0=not BOND member. |
| *corr; add* | Correspondence and address details |
| *activities* | The trustees’ description in their own words of what they do and who they help |

In addition, the ‘collated file’ includes a series of 201 country binary variables (1: charity operates in that country; 0: charity does not operate in that country).

Each file is sorted, with the biggest organisations (as measured by 2012 headline income) at the top.

**Licence**

These files are based on the analysis of data provided by the Charity Commission, are subject to Crown database copyright or Crown Copyright and contain public sector information licensed under the terms of the Open Government licence v2.0. All necessary legal requirements relating to its use and re-use have been complied with.

**References**

Alkire, S., Conconi, A. and Seth, S. (2014), *Multidimensional Poverty Index 2014: Brief Methodological Note and Results*, Oxford: OPHI.

Department for International Development (DFID) (2011), *Bilateral Aid Review: Technical Report*, London: DFID.

Hénon, S. (2014), *Measuring Private Development Assistance: Emerging Trends and Challenges*, Bristol: Development Initiatives.

Kaufmann, D., Kraay, A. and Mastruzzi, M. (2011), 'The Worldwide Governance Indicators: Methodology and Analytical Issues', *Hague Journal on the Rule of Law*, 3: 02, 220-246.

Mayer, T. and Zignago, S. (2011), *Notes on Cepii’s Distances Measures: The Geodist Database*: CEPII Document de Travail No 2011-25.

Office for National Statistics (ONS) (2013), *Qs213ew: 2011 Census: Country of Birth (Expanded), Regions in England and Wales*, Titchfield: Office for National Statistics.

Pollet, I., Habraken, R., Schulpen, L. and Huyse, H. (2014), *The Accidental Aid Worker. A Mapping of Citizen Initiatives for Global Solidarity in Europe*, Leuven: HIVA-KU.

Schnable, A. (2014) ‘Exporting Bootstraps: Aid Narratives and Grassroots NGOs’, *Center for the Study of Social Organisation Working Paper 8*.

United Nations (2011), *State of the World's Volunteerism Report*, Bonn: United Nations Volunteers.

World Bank (2012), *Country and Lending Groups. Available from* [*Http://Data.Worldbank.Org/About/Country-and-Lending-Groups*](Http://Data.Worldbank.Org/About/Country-and-Lending-Groups).

World Bank (2013), *Population Statistics by Country. Available from* [*Http://Databank.Worldbank.Org/Data/Databases/Population-Dynamics*](Http://Databank.Worldbank.Org/Data/Databases/Population-Dynamics).
